# Changes in the disease-specific quality of life following Dor fundoplication. A multicentre cross-sectional study

**DOI:** 10.1016/j.amsu.2020.05.018

**Published:** 2020-05-28

**Authors:** Guo Hou Loo, Reynu Rajan, Mahadevan Deva Tata, Nik Ritza Kosai

**Affiliations:** a Surgical Trainee, Department of General Surgery, Faculty of Medicine, Universiti Kebangsaan Malaysia Medical Centre, Jalan Yaacob Latiff, Bandar Tun Razak, 56000, Kuala Lumpur, Malaysia; b Consultant Bariatric Surgeon Department of Surgery, Faculty of Medicine, Universiti Kebangsaan Malaysia Medical Centre, Jalan Yaacob Latiff, Bandar Tun Razak, 56000, Kuala Lumpur, Malaysia; c Consultant Upper Gastrointestinal & Bariatric SurgeonDepartment of Surgery, Tuanku Ja'afar Hospital Jalan Rasah, Bukit Rasah, Seremban, 70300, Negeri Sembilan, Malaysia; d Consultant Upper Gastrointestinal & Bariatric Surgeon, Head of Unit of Upper Gastrointestinal and Minimally Invasive Surgery, Department of Surgery, Faculty of Medicine, Universiti Kebangsaan Malaysia Medical Centre, Jalan Yaacob Latiff, Bandar Tun Razak, 56000, Kuala Lumpur, Malaysia

**Keywords:** Anti-reflux surgery, Asian population, GERD HRQL questionnaire, Refractory GERD, VISICK Score

## Abstract

**Background:**

Gastrooesophageal reflux disease (GERD) is a spectrum of symptoms arising from the laxity of the cardio-oesophageal junction. Anti-reflux surgery is reserved for patients with refractory GERD. Anterior partial fundoplication (Dor) is a regularly performed anti-reflux surgery in Malaysia. We intend to determine the improvement in disease-specific quality of life in our patients after surgery.

**Methods:**

A multicentre cross-sectional study was conducted to assess patients’ improvement in disease-specific quality of life after Dor fundoplication. Ethics approval was obtained from our institutional review board. Patients between the ages of 18 and 65 years who underwent Dor fundoplication within the past five years were assessed using the GERD HRQL as well as the VISICK score via telephone interview. We excluded cases of revision surgery.

**Results:**

Out of 129 patients screened, 55 patients were included. We found a significant improvement in patients’ GERD HRQL score with the pre-operative mean score of 28.3 ± 9.39 and 6.55 ± 8.52 post-operatively, *p* < 0.01.50.9% of patients reported a VISICK score of 1. However, we noticed a deterioration in the GERD HRQL and VISICK score in patients followed up four years after surgery. This consisted of 25.5% of total patients.

**Conclusion:**

Dor Fundoplication improves the overall disease-specific quality of life in patients with refractory GERD in the short term period. Recurrence of symptoms causing a deterioration in the quality of life is seen in patients followed up beyond four years of index surgery.

## Highlights

• A multicenter cross-sectional study was conducted to assess patients’ disease-specific quality of life after Dor fundoplication.
• The mean GERD HRQL score pre-operatively was 28.3 ± 9.39 and improved to a score of 6.55 ±8.52 post-operatively.
• 52.7% of patients do not require PPIs after surgery, while 23.6% still required PPI rarely (<1/week).
• The GERD HRQL global satisfaction score showed that 81.8% of patients were satisfied with their symptoms post-operatively.
• Symptom recurrence after four years and beyond may cause a deterioration in the disease-specific quality of life.

## 1 Introduction

The passage of stomach contents into the oesophagus is a physiological process, termed gastrooesophageal reflux. This becomes pathological when is causes distressing symptoms, or there is evidence of macroscopic damage [1]. Early studies in Europe have shown that GERD has widespread reaches. A GERD prevalence study conducted showed that as much as 20% of the Western European is affected [2]. The prevalence of GERD in the Asian population was studied in two separate studies in Singapore, and it showed a four-fold increase, from 1.6% in 1994 to 10.6% in 2001 [3], [4].

GERD occurs more commonly in patients with hiatus hernia, in those who have lower oesophageal sphincter dysfunction and those with impaired oesophageal acid clearance or delayed gastric emptying. The diagnosis of GERD is usually clinical, as it involves the perception of symptoms by the patient [1]. Several questionnaires have been used for the diagnosis of GERD, such as GerdQ, which was developed for the diagnosis of GERD in the primary care setting [5]. An upper endoscopy is not required for the diagnosis or treatment of GERD, but it is essential to rule out malignancies or complications related to GERD [5].

Despite treatment with proton pump inhibitors (PPIs), some patients will have persistent symptoms. In a systematic review, 45% of patients on PPIs in observational studies reported having persistent reflux symptoms [6]. Anti-reflux surgery is usually reserved for patients who have an oesophageal mucosal injury (complications of reflux) despite maximal medical therapy, persistent reflux symptoms despite medical therapy, and those who are unable to tolerate long term medical therapy [7].

First described by Rudolph Nissen in 1955, Nissen's fundoplication has been the mainstay of surgery for patients with refractory GERD disease since [8]. This surgery involves a 360° wrapping of the distal oesophagus with the fundus of the stomach, usually performed transabdominally [8]. This surgery is valid and has been proven to be able to control reflux symptoms in 91% of the patients over ten years [9]. The laparoscopic approach showed that symptom control is equivalent to an open approach, with less risk of incisional hernias [10], [11]. Modifications to the original Nissen fundoplication was devised due to complications of a total wrap such as dysphagia, gas bloatedness and inability to belch [12]. A partial 270-degree posterior wrap (Toupet) and a 180° anterior partial wrap (Dor) has been shown to be equally effective when compared to Nissen fundoplication in reflux symptom control over the long term, with lower rates of dysphagia [12], [13].

Patient's disease-specific quality of life after fundoplication has been assessed in a randomised controlled trial comparing Nissen and Dor fundoplication over two years. The authors concluded that both methods were comparable in terms of post-operative quality of life[14]. A cross-sectional study which assessed the patients' quality of life after Dor fundoplication over a mean follow up period of 52months concluded that Dor fundoplication could lead to satisfying long-term symptom control. Both of these studies, however; was done in the western population, by the same author. In 2010, a cross-sectional study was done by Kumaresan et al. assessing the quality of life after Dor fundoplication. 25 patients were followed up over a median of nine months, and the overall satisfaction rate was 84% [15]. We intend to assess patients who have underwent Dor fundoplication over a more extended period to gauge whether the improvement in disease-specific quality of life persists in the long term.

## 2 Materials and methods

A multicentre cross-sectional study was conducted to assess patients' improvement in disease-specific quality of life after Dor fundoplication. Patients were recruited from two upper gastrointestinal centres in Malaysia with one centre being a tertiary referral hospital and the other a teaching university hospital. The surgery was performed by upper gastrointestinal surgeons with more than five years’ experience. We perform a routine repair of the hiatal opening with fixation of the mobilised oesophagus using non-absorbable sutures, with anterior partial fundoplication as described by Watson et al. [16].

The study was conducted in accordance with the principles of the Declaration of Helsinki. Ethics approval was obtained before the start of this study from our institutional review board. This study was also registered with the Thai Clinical Trials Registry. Written informed consent was taken before study commencement. All data collected were kept confidential, and patients were allowed to refuse participation in the study. Data presented do not identify individuals.

A purposive sampling method was employed. All patients between the ages of 18 and 65years who underwent Dor fundoplication between January 1, 2012 until January 31, 2017 were contacted via a telephone call. We excluded patients who had a revision surgery upon presentation to the two centres, and patients who had more than one procedure performed simultaneously.

These patients were then read a pre-prepared script, a verbal consent, and an explanation regarding the nature of the study. GERD Health-Related QoL (HRQL), a validated disease-specific quality of life questionnaire and VISICK score were used for evaluation. The questionnaires were then matched with the pre-operative values taken from in the patients’ medical records. Statistical significance was set at *p* < 0.05. Variables with normal distribution were expressed as mean ± SD, the significance of differences before and after surgery was evaluated with a paired *t-test*. Data were analysed using the SPSS statistical software version 24, IBM® (New York, United States). This work has been reported in line with the STROCSS criteria [17].

## 3 Results

Out of 129 patients screened, 55 patients agreed to be interviewed. Of the 74 patients who were excluded, 36 patients were not contactable, and 38 patients declined to be interviewed for a variety of reasons. Of the patients included, 78.2% (n = 43) were operated at a teaching university hospital, and the remaining were operated at a tertiary referral hospital. The majority of patients recruited were of Malay ethnicity (76.4%), and 58.2% were male patients. The mean age was 44.1 ± 9.7 years. The mean follow-up time in years was 2.84 ± 1.21 years. Baseline characteristic of patients was summarised in Table 1.

**Table 1:** Baseline characteristic of study participants.

Variables (n = 55)	
Mean age±SD (years)	44.1 ± 9.7
Gender no (%) Male Female	32 (58.2%) 23 (41.8%)
Ethnicity, no (%) Malay Chinese Indian	42 (76.4) 5 (9.1) 8 (14.5)
Mean weight±SD (kgs) Before surgery After surgery	85.67 ± 26.44 69.10 ± 22.76
Mean follow up time±SD (years)	2.84 ± 1.21
Range of follow up time (years)	1–6
PPIs usage after surgery no (%) i) Do not require PPIs ii) Require PPIs rarely (<1/week) iii) Require PPIs at least once/week iv) Require daily PPIs	29 (52.7) 13 (23.6) 8 (14.5) 5 (9.1)

*PPI= Proton Pump Inhibitors, SD = standard deviation.

The GERD HRQL can be divided into a heartburn score, a dysphagia score, and a general quality of life score. All of these scores had shown statistically significant improvement post-operatively**.** The mean GERD HRQL score pre-operatively was 28.3 ± 9.39 and improved to a score of 6.55 ± 8.52 post-operatively *p* < 0.01. The pre-operative GERD HRQL heartburn score improved from 20.71 ± 7.96 to 4.29 ± 5.79 post-operatively, *p* < 0.01, while the dysphagia score improved from 4.87 ± 3.04 to 1.65 ± 2.33 post-operatively, *p* < 0.01.52.7% of patients do not require PPIs after surgery, while 23.6% still required PPI rarely (<1/week). This is illustrated in Table 2. The GERD HRQL global satisfaction score showed that 81.8% of patients were satisfied with their symptoms post-operatively, in contrast to -pre-operatively, where all the patients were either neutral or dissatisfied with their symptoms.

**Table 2:** GERD HRQL scores before and after surgery, with p-value.

Question	Before Surgery (mean ± SD)	After Surgery (mean ± SD)	p-value
Total heartburn scores	20.71 ± 7.96	4.29 ± 5.67	p < 0.01
Total dysphagia score	4.87 ± 3.04	1.65 ± 2.33	p < 0.01
Do you have bloatedness?	3.31 ± 1.32	1.16 ± 1.31	p < 0.01
Does taking medication affect your life?	2.85 ± 1.28	0.62 ± 1.147	p < 0.01
Total GERD HRQL Scores	28.44 ± 9.39	6.55 ± 8.52	p < 0.01

*SD = standard deviation.

**Graph 1:**
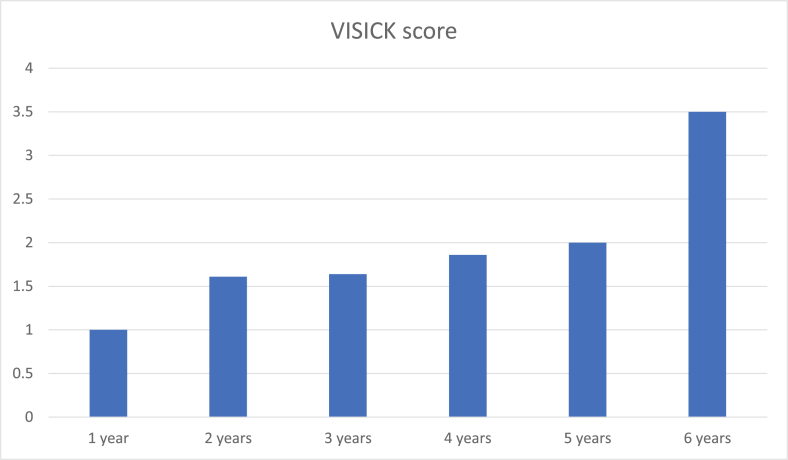
Worsening trend of VISICK score over time.

The mean VISICK score was 1.73 ± 0.91.83.6% of patients reported a post-operative VISICK score of 1 and 2 (Table 3). However, the VISICK score deteriorated with time, especially four years and beyond (graph 1).

**Table 3:** Post-operative VISICK scores of patients.

VISICK score	Frequency (n)	Percentage (%)
**1**	28	50.9
**2**	18	32.7
**3**	5	9.1
**4**	4	7.3

## 4 Discussion

Anti-reflux surgery intends to re-establish the function of the lower oesophagal sphincter and improve lower oesophagal motility[18]. Nissen fundoplication, a 360° wrap, is an effective treatment in the medium and long-term follow up in most patients[19]. However, post-operative dysphagia is a troublesome adverse effect, with an incidence of up to 23% [20]. A previous randomised study demonstrated that at six-months follow up; Dor fundoplication achieved effective reflux symptom control with reduced dysphagia and gas-related symptoms [21]. At five years follow up of the randomised controlled trial, Dor fundoplication is favoured in overall outcome compared to Nissen fundoplication(12).

Disease-specific quality of life after Dor fundoplication showed a significant improvement in our patients over the short and medium-term follow up. These findings are similar to a study performed on the western population done by Raue et al. [14]. VISICK score is a non-specific scoring system, but it provides a gauge of the effectiveness of surgery. Recurrence of reflux symptoms after Dor fundoplication can usually be managed with PPIs alone, without resorting to revision surgery.

The recurrence of reflux symptoms over time may reflect the more inferior disease-specific quality of life ratings by patients. As this deterioration in score might have been due to weight gain over time, we took that into account as well [22]. In our study, we found that there were no significant differences in mean weight of patients before surgery (mean 85.67 ± 26.44 kgs) and after surgery (mean 69.10 ± 22.76) *p* = 0.43.

A large clinical outcome study by Chen Zhen et al. showed that even after 10years after Dor fundoplication (n = 89), overall satisfaction with the outcomes of surgery remains stable. They also reported 89.8% of their patients considered their decision to undergo anti-reflux surgery to be correct. With regards to the use of PPIs after surgery, our study showed a better result, with just 23.6% still requiring PPI, while they reported 38.4% of their patients still require an antisecretory medication after surgery [23].

Our study has several limitations. Limited sample size in our study is due to a high dropout rate (27.9%) as patients do not come for follow-up several years after surgery. We are unable to postulate the reason for this high drop-out rate, but it is possible that due to the long study period, patients were lost to follow-up. Secondly, we do not have a control group to compare the improvement in disease-specific quality of life. A randomised controlled trial comparing disease-specific quality of life after Dor fundoplication compared to other anti-reflux surgeries will provide a more objective result. A network meta-analysis comparing various anti-reflux surgeries in fifty-one randomised controlled trials concluded posterior partial fundoplication provides the best balance of long-term, durable reflux control with less dysphagia [24].

## 5 Conclusion

Dor fundoplication is a reliable anti-reflux surgery which is shown to improve disease-specific quality of life in the short and middle term. Symptom recurrence after four years and beyond may cause a deterioration in the quality of life. However, this usually can be managed with medication alone. The majority of patients report that they are satisfied with their symptoms post-operatively. A randomised controlled trial comparing quality of life after Dor fundoplication with other anti-reflux surgeries is required.

## Source of funding

None.

## Provenance and peer review

Not commissioned, externally peer reviewed.

## Declaration of competing interest

The authors declare that they have no conflict of interest.
